# β_1_-Adrenoceptor Autoantibodies from DCM Patients Enhance the Proliferation of T Lymphocytes through the β_1_-AR/cAMP/PKA and p38 MAPK Pathways

**DOI:** 10.1371/journal.pone.0052911

**Published:** 2012-12-31

**Authors:** Yunhui Du, Li Yan, Jin Wang, Wenzhang Zhan, Kai Song, Xue Han, Xiao Li, Jimin Cao, Huirong Liu

**Affiliations:** 1 Department of Physiology and Pathophysiology, School of Basic Medical Sciences, Capital Medical University, Beijing, China; 2 Department of Marine Bioengineering, Marine College, Shandong University, Weihai, Shandong, China; 3 Department of Physiology and Pathophysiology, Institute of Basic Medical Sciences, Peking Union Medical College, Beijing, China; 4 Department of Physiology, Shanxi Medical University, Taiyuan, Shanxi, China; 5 Department of Internal Medicine, General Hospital of Tonghua Mining Group CO. LTD, Baishan, Jilin, China; 6 Department of Internal Medicine, A Peace Hospital Attached to Changzhi Medical College, Changzhi, Shanxi, China; 7 The Key Laboratory of Remodeling-related Cardiovascular Diseases, Capital Medical University, Ministry of Education, Beijing, China; University of Torino, Italy

## Abstract

**Background:**

Autoantibodies against the second extracellular loop of the β_1_-adrenergic receptor (β_1_-AA) not only contribute to increased susceptibility to heart failure, but also play a causative role in myocardial remodeling through their sympathomimetic-like effects that are induced upon binding to the β_1_-adrenergic receptor. However, their role in the function of T lymphocytes has never been previously investigated. Our present study was designed to determine whether β_1_-AA isolated from the sera of dilated cardiomyopathy (DCM) patients caused the proliferation of T cells and the secretion of cytokines.

**Methods:**

Blood samples were collected from 95 DCM patients as well as 95 healthy subjects, and β_1_-AA was detected using ELISA. The CD3^+^T lymphocytes were selected separately through flow cytometry and the effect of β_1_-AA on T lymphocyte proliferation was examined by CCK-8 kits and CFSE assay. Western blotting was used to analyze the expressions of phospho-VASP and phospho-p38 MAPK.

**Results:**

β_1_-AA enhanced the proliferation of T lymphocytes. This effect could be blocked by the selective β_1_-adrenergic receptor antagonist metoprolol, PKA inhibitor H89, and p38 MAPK inhibitor SB203580. Furthermore, the expression of the phosphorylated forms of phospho-VASP and phospho-p38 MAPK were markedly increased in the presence of β_1_-AA. β_1_-AA also inhibited the secretion of interferon-γ (IFN-γ) while promoting an increase in interleukin-4 (IL-4) levels.

**Conclusions:**

These results demonstrate that β_1_-AA isolated from DCM patients binds to β_1_-AR on the surface of T cells, causing changes in T-cell proliferation and secretion through the β_1_-AR/cAMP/PKA and p38 MAPK pathways.

## Introduction

Dilated cardiomyopathy (DCM), a heart condition characterized by left ventricular dilation and progressive loss of cardiac function, represents the main cause of severe heart failure in younger adults and thus is a challenge for public health [1]. About one-third of DCM cases are genetic in origin, whereas the etiology of the remaining 70% is poorly understood [2]. Autoimmune responses against various myocardial antigens have been proposed to play an important role in the triggering or progression of DCM [3]–[4]. However, the mechanisms involved in its pathological process have not been elucidated.

Recent studies have reported that the activation of T lymphocytes and an increase in inflammatory cytokines are involved in chronic heart failure due to DCM [5], [6]. A role for altered T cell proliferation was indicated by our previous studies which reported elevated ratios of CD4^+^/CD8^+^T lymphocytes during heart failure in rats [7]. These researches suggest that some certain elements may contribute to T lymphocyte disorder in the pathogenesis of chronic heart failure.

Evidences suggest that antigens newly exposed to the immune system upon cardiac damage trigger a myocardial autoimmune response, leading to ventricular remodeling and further damage to the myocardium [8]. In the 1990s, investigators reported that the autoimmune antibody (β_1_-AA) against the second extracellular loop (β_1_-AR-EC_II_ amino acid residues 197–223, 100% sequence identity between humans and rats [9]) of the β_1_-adrenergic receptor is present in the sera of patients with cardiovascular diseases [10]–[13]. This led to the proposal stating that β_1_-AA acts similarly to a β_1_-adrenergic receptor agonist based on findings of an increased beating rate in neonatal rat cardiomyocytes [14]. We have reported that the long-term presence of β_1_-AA led to myocardial damage with increased ratios of CD4^+^/CD8^+^ T lymphocytes [15]. These clinical and experimental results, taken together with the expression of β_1_-AR on the surface of T lymphocytes [16], strongly suggest that β_1_-AA might mediate T cell abnormalities in chronic heart failure patients. However, it is not known if the β_1_-AA isolated from DCM patients, which mimics the action of catecholamine, could recognize the cognate receptor and interfere with T lymphocytes.

Therefore, the aims of the current study were as follows: (1) to observe the effects of β_1_-AA on the proliferation and secretion of T cells, and (2) to identify the signaling pathways that mediate T cell responses.

## Materials and Methods

### Materials

Metoprolol (selective β_1_-adrenergic receptor antagonist), isoproterenol (β_1_/β_2_-adrenergic receptor agonist), SB203580 (selective p38 MAPK inhibitor), and H89 (selective PKA inhibitor) were purchased from Sigma-Aldrich Chemicals Company (USA). Carboxyfluorescein diacetate succinimidyl ester (CFSE) was purchased from Invitrogen™ (Life Technologies Corporation, Belgium). Polyclonal antibodies directed against phospho-VASP (Ser157), phospho-p38 MAPK (Thr180/Tyr182), VASP, and p38 MAPK were obtained from Cell Signaling Technologies (USA). All chemicals utilized in this study were of analytical grade.

### Animals

Healthy male 8-week-old Sprague–Dawley rats, with normal blood pressure and heart rate, weighing 200 g to 240 g, were selected for this study. The experimental procedures were conducted in adherence to the “Guiding Principles in the Use and Care of Animals” published by the National Institutes of Health (NIH Publication No. 85-23, Revised 1996), the Guide for the Care and Use of Laboratory Animals protocol, published by the Ministry of the People’s Republic of China (issued on 3 June 2004), and approved by the Institutional Animal Care and Use Committee of Capital Medical University.

### Patients and Samples

The study adheres to the principles of the Declaration of Helsinki and Title 45, U.S. Code of Federal Regulations, Part 46, Protection of Human Subjects, Revised 13 November 2001, effective 13 December 2001. Ninety-five chronic heart failure patients were recruited from the Air Force General Hospital of the People’s Liberation Army and General Hospital of Tonghua Mining Group Co., LTD, all of which were suffering from dilated cardiomyopathy (DCM) (New York Heart Association functional class II to IV), with a left ventricular diastolic volume >110 ml/m^2^ and an ejection fraction <45% (by echocardiography). DCM was diagnosed when coronary heart diseases were excluded by angiography and exposures to cardiotoxic substances, myocarditis, or other systemic heart diseases were not evident from clinical history. In ventriculography, all patients exhibited a diffuse reduction in wall motion. At the time of sample acquisition, all patients were stable under therapy with diuretics, ACE inhibitors, digitalis, and nitrates. The control group consisted of 95 healthy subjects randomly selected from the same community with normal clinical, ECG, and echocardiography examinations. On the basis of the resulting measurements of β_1_-AA, the DCM patients were divided into a β_1_-AA-positive group (*n* = 44) and a β_1_-AA-negative group (*n* = 51). Clinical characteristics are summarized in Table 1 and Table 2. Venous blood samples were collected without an anticoagulant. After centrifugation at 4°C, the serum was immediately separated and stored at −80°C until assay.

**Table 1:** Clinical characteristics of patients with chronic heart failure due to DCM (mean ± SD).

	β_1_-AA-positivegroup(n = 44)	β_1_-AA-negativegroup(n = 51)	Healthygroup(n = 95)
Age (year)	62±12	59±6	56±10
Gender (male/female)	25/19	27/24	53/42
Duration of illness (year)	6.38±4.26	6.17±3.74	−
NYHA	3.6±0.8	3.2±0.7	−
LVEF (%)	35.3±6.1**	41.5±7.0**	69.4±6.6
LVEDD (mm)	67.5±9.6**	63.8±7.7**	46.5±8.3
LVESD (mm)	57.6±8.6**	55.3±7.9**	32.6±7.3
Medications			
Diuretics (%)	52 (23/21)	59 (30/21)	−
Digoxin (%)	50 (22/22)	57 (29/22)	−
ACE-inhibitors (%)	100 (44/0)	98 (50/1)	−
β-Blockers (%)	45 (20/24)	37 (19/32)	−
Anti-arrythmia agents (%)	23 (10/34)	31 (16/35)	−

*DCM* dilated cardiomyopathy; *NYHA* New York Heart Association; *LVEF* left ventricular ejection fraction; *LVEDD* left ventricular end-diastolic diameter; *LVESD* left ventricular end-systolic diameter. Values are expressed as mean ± SD or number (%) of patients. **P<0.01 *versus* healthy group.

**Table 2:** Holter Electrocardiographic Findings (mean ± SD).

	β_1_-AA-positivegroup(n = 44)	β_1_-AA-negativegroup(n = 51)
Atrial fibrillation (%)PVCs	34 (15/29)	27 (14/27)
PVCs/24 h	2,136±4,340	1,564±2,674
Multiform PVCs (%)VT	82 (36/8)**	63 (32/19)
Presence of VT (%)	65 (24/20)**	43 (22/29)
Maximal runs of VT (beats)	7±5	6±4
Longest VT duration (s)	2.2±1.4	2.7±1.2
Fastest VT rate (beats/min)	187±32*	145±24

*PVCs* premature ventricular contractions; *VT* ventricular tachycardia. Values are expressed as mean ± SD or number (%) of patients.

* *P*<0.05,

** *P*<0.01 β_1_-AA-positive group *versus* β_1_-AA-negative group.

The Institutional Committee for the Protection of Human Subjects of Capital Medical University approved this research protocol. All patients were informed of the purpose and protocol of the investigational nature of the study. Both oral informed consent and written consent were obtained.

### Peptide Synthesis

The peptide corresponding to the sequence (amino acid residues 197–223) of the second extracellular loop of the human β_1_-AR [15]: H-W-W-R-A-E-S-D-E-A-R-R-C-Y-N-D-P-K-C-C-D-F-V-T-N-R-C was synthesized using an automated peptide synthesizer by solid-phase methods. Peptide purity was judged by high-performance liquid chromatography (HPLC) using an automated amino-acid analyzer. Peptide preparations were 98% pure as judged by analytical HPLC. This work was performed by a contractor (Qiang Yao, Shanghai Bio Scientific Commercial Development Co. Ltd., China).

### Enzyme-linked Immunosorbent Assay (ELISA)

The titer of β_1_-AA was measured by ELISA, and the results are expressed as optical-density (OD) units according to published methods [17]. Briefly, the synthetic peptide described above (5 mg/ml) in 100 mmol/l Na_2_CO_3_ (pH 11.0), was coated onto the wells of microtiter plates overnight at 4°C. The wells were then saturated with 0.1% PMT buffer [0.1% (w/v) albumin bovine V, 0.1% (v/v) Tween-20 in phosphate-buffered saline (PBS), pH 7.4] for 1 h at 37°C. After washing 3 times with PBS-T, serial dilutions of human sera were added for 1 h at 37°C. After 3 washings, biotinylated goat-antihuman IgG antibodies (Sigma) (1∶1000 dilutions in PMT) were added for 1 h at 37°C. After 3 washings, streptavidin-peroxidase conjugate (Sigma) at 1∶2000 dilution in the same buffer was added to the wells and incubated under the same conditions. Finally, 2, 2-azino-di (3-ethylbenzothiazoline) sulfonic acid (ABTS)-H_2_O_2_ (Roche, Switzerland) substrate buffer was added and reacted for 30 min in the dark at room temperature. The OD values were measured at 405 nm using a microplate reader (Spectra Max Plus, Molecular Devices, USA). We also calculated antibody titer according to the ratio (P/N) of OD values of positive/negative controls [(specimen OD-blank control OD)/(negative control OD-blank control OD)] [7]. Control samples were prepared as follows: 95 sera samples from healthy humans with an OD value of less than 2.5 times the background OD were pooled and centrifuged at 1,500 rpm for 10 min, and the supernatants were then divided into small aliquots and stored for subsequent use. Samples positive or negative for β_1_-AA were defined as P/N ≥2.1 or P/N ≤1.5, respectively.

### Preparation of Immunoglobulin G

Immunoglobulin G fractions (IgG) from the sera of 44 β_1_-AA-positive or from 51 β_1_-AA-negative DCM patients were prepared by MabTrap Kit (Amersham Bioscience, Sweden). The concentrations (µg/ml) and specificities of purified IgGs were determined by the Bicinchoninic Acid (BCA) Protein Assay (Pierce, USA) and ELISA, respectively.

### Isolation and Culture of CD3^+^ T Cells

Rats were anesthetized with ether, and a blood sample was taken from the abdominal aorta. Mononuclear cells were prepared from the freshly drawn blood samples by Ficoll-Hypaque (1.077 g/l) density gradients. Whole blood (40 ml) was collected with heparin and centrifuged. After the plasma was discarded, white cells and erythrocytes were taken and suspended in 10 ml of PBS (pH 7.4). These suspensions were added to 5 ml of Ficoll-Hypaque and then centrifuged at 2,000 rpm for 20 min. Mononuclear cells were collected, washed twice with PBS, and centrifuged at 1,500 rpm for 10 min. To eliminate adherent cells (monocytes), cell suspensions were placed into culture flasks with 5 ml of Roswell Park Memorial Institute medium 1640 (RPMI) with gentamicin (100 µg/ml), L-glutamine (2 mmol/l), and 10% fetal calf serum (Invitrogen, USA). Cells were incubated at 37°C in humidified air containing 5% CO_2_ for 30 min. Nonadherent cells were collected, washed, and isolated by centrifugation at 1,500 rpm for 10 min and suspended in culture medium. CD3^+^T cells were selected from the mononuclear cells using a flow cytometer (BD Biosciences, USA).

### Immunofluorescence Staining

CD3^+^T cells were gently washed with PBS (pH 7.4) and immediately fixed with 4% paraformaldehyde (w/v) for 20 min. Cells were blocked in PBS containing 5% bovine serum albumin (BSA) (w/v). The cells were then incubated overnight at 4°C with the IgG fractions (25 µg/ml) from β_1_-AA-positive DCM patients at a 1∶500 dilution. Following three PBS washes, cells were incubated in donkey anti-human IgG tagged with fluorescein isothiocyanate (FITC) as the secondary antibody for 1 hour in the dark at 37°C. After being rinsed with PBS, cover slips with mounting medium containing 4′, 6-diamidino-2-phenylindole (DAPI) stain nuclei were coated. Negative controls were performed by omitting primary antibodies. Images were acquired using a Zeiss 510 Meta Confocal microscope (63 power oil 1.40 NA (Zeiss, Germany), pinhole equals 1.0 Airy Disc) using the Carl Zeiss Imaging software.

### Culture of Beating Neonatal Cardiomyocytes

Hearts were removed aseptically from 1 to 2-day-old Sprague–Dawley rats and cultured as described [7]. The number of beats of a selected isolated myocardial cell or a cluster of synchronously contracting cells in each of 10 fields was counted for 15 s each. The IgG fractions from β_1_-AA-positive DCM patients and corresponding receptor agonists were added, and the cells were observed for 5 min after each addition. This procedure was repeated three times in different cultures to yield results representing a total of 30 cells or cell clusters. The basal beating rate was 145±15 beats/min.

### CD3^+^T Cell Proliferation Assays

#### 1. CCK-8 assay

CD3^+^T cells (5×10^5^ cells/ml) were cultured for 48 h with or without mitogens in either the presence or absence of β_1_-AA (12.5 µg/ml, 25 µg/ml, and 50 µg/ml), H89 (1 µmol/l), metoprolol (1 µmol/l), isoproterenol (0.1 µmol/l), and SB203580 (1 µmol/l). The mitogens used were 3 µg/ml soluble mouse anti-rat CD3 mAb (Clone 1F4, Biolegend, USA) and 1 µg/ml soluble mouse anti-rat CD28 mAb (Clone JJ319, Biolegend). Agonists were added to cell suspensions together with the mitogens while the antagonists were added 1 h before the agonists. After each treatment, 10 µl CCK-8 solution was added to each well, and the cells were incubated for 4 hours at 37°C. The absorbance at 450 nm was measured using a microplate reader with the wavelength correction set to 630 nm.

#### 2. CFSE-labeling of lymphocytes

CFSE (10 mmol/l in DMSO (Invitrogen) was diluted in PBS. CD3^+^T lymphocytes were suspended in PBS supplemented with 0.05% BSA and 4 µmol/l CFSE (2×10^7^ cells/ml) for 10 min at 37°C in a 5% CO_2_ atmosphere. Cells were washed, diluted in 0.5 ml culture medium and incubated for 30 min at 37°C in 5% CO_2_ to stabilize the CFSE-labeling. The efficiency of labeling untreated cells was >95%.

### Analysis of cAMP Production

T lymphocytes were washed twice in Tris buffer containing 120 mmol/l NaCl, 1 mmol/l MgCl_2_, 5 mmol/l KCl, 0.6 mmol/l CaCl_2_, 25 mmol/l Tris (hydroxymethyl-amino-ethane), 5 mmol/l glucose, and 0.1 mmol/l human albumin, adjusted to pH 7.4 with HCl. Cells were suspended in RPMI 1640/FBS medium to a final density of 2×10^6^ cells/ml. The samples were incubated with 1-methyl-3-isobutylxanthine (0.5 mmol/l) for 10 min and then stimulated for 10 min with anti-rat CD3/CD28 mAb in either the absence or presence of the β_1_-AA or agonist, with or without an antagonist. Reactions were terminated by adding 2 N HCl-0.1 mol/l EDTA followed by incubating the samples at 80°C for 10 min. After centrifugation of the precipitated protein, the samples were neutralized with CaCO_3_ and cAMP was measured using an enzyme immunoassay (Biotrak, Amersham, UK) as specified by the manufacturer. The cAMP concentrations are expressed as pg/ml.

### Analysis of VASP-Ser157 Phosphorylation, p38 MAPK Phosphorylation, Total VASP, and Total p38 MAPK

Cells were lysed in PRO-PREP protein extract solution. The sample was centrifuged at 10,000 rpm for 20 min at 4°C. Protein concentration was determined by the BCA assay (Pierce). An equal volume of 2× SDS sample buffer (0.1 mol/l Tris-Cl, 20% glycerol, 4% SDS, and 0.01% bromophenol blue) was added to an aliquot of the supernatant fraction from the lysates, and the mixture was boiled for 5 min. Aliquots of 30 µg of protein were subjected to 10% SDS-polyacrylamide gel electrophoresis for 1 h 30 min at 110 V. The separated proteins were transferred to PVDF membranes for 2 h at 20 mA with a SD Semi-dry Transfer Cell (Bio-Rad). The membranes were blocked with 5% nonfat milk in Tris-buffered saline containing 0.05% Tween 20 (TBS-T) for 2 h at room temperature. The membranes were then incubated with rabbit polyclonal anti-rat phospho-VASP (Ser 157), anti-rat VASP, anti-rat phosphor-p38 MAPK, and anti-rat p38 MAPK all diluted at 1∶1000 in 5% nonfat milk in TBS-T overnight at 4°C, and then the bound antibody was detected using horseradish peroxidase-conjugated anti-rabbit IgG. The membranes were washed and proteins detected using a Western Blotting Luminol Reagent system and autoradiography.

### Analysis of Cytokine Production

IFN-γ and IL-4 levels in culture supernatants were analyzed using a commercial ELISA kit (R&D Systems, USA), according to the manufacturer’s protocol. The absorbance at 450 nm was measured using a microplate reader with the wavelength correction set to 630 nm.

### Statistical Analysis

Values are expressed as means ± standard deviation (SD). Statistical analysis was performed with SPSS 13.0 software. The student *t*-test was used to compare two independent sample means, and one-way ANOVA was used to compare the means of more than two samples. A value of *p*<0.05 was considered statistically significant.

## Results

### Serum Levels of β_1_-AA were Markedly Increased in DCM Patients Compared with Healthy Subjects

**Figure 1:**
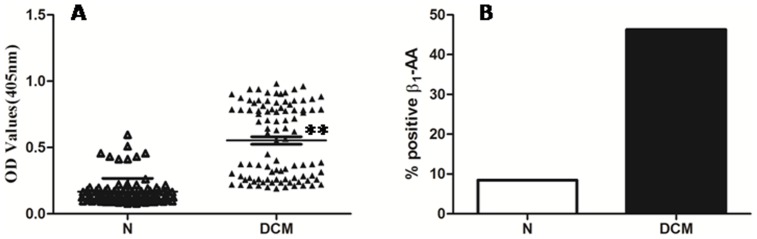
Titers and incidence of β_1_-AA in 95 healthy subjects and 95 patients with DCM. **A.** Titers of β_1_-AA from 95 healthy subjects (*open squares*) and 95 patients with DCM (*filled squares*). Scatter plot represents the titers of β_1_-AA for each patient in each group. Experiments were repeated twice per sample. **B.** Percentage of β_1_-AA-positive sera from two different groups. *N,* normal group, *DCM* dilated cardiomyopathy. **p<0.01 versus N group.

We tested for the presence of autoantibodies directed against the second extracellular loop of β_1_-AR by ELISA in sera collected from 95 patients with chronic heart failure due to DCM and from 95 control subjects. Subjects’ clinical data are summarized in Table 1 and Table 2. There was no difference in age or gender distribution. Compared with healthy individuals, serum titers of β_1_-AA were markedly increased in DCM patients (0.553±0.028 *vs.* 0.167±0.0102, *p*<0.01) (Fig. 1A). As illustrated in Fig. 1B, only 8 of 95 healthy subjects were β_1_-AA positive (8.42%), whereas 44 of 95 DCM patients had a P/N value greater than 2.1 (46.3% positive). These data demonstrate that serum levels of β_1_-AA were markedly increased in DCM patients with heart failure when compared with healthy subjects.

### β_1_-AA Bound to β_1_-ARs on the Surface of CD3^+^T Cells

**Figure 2:**
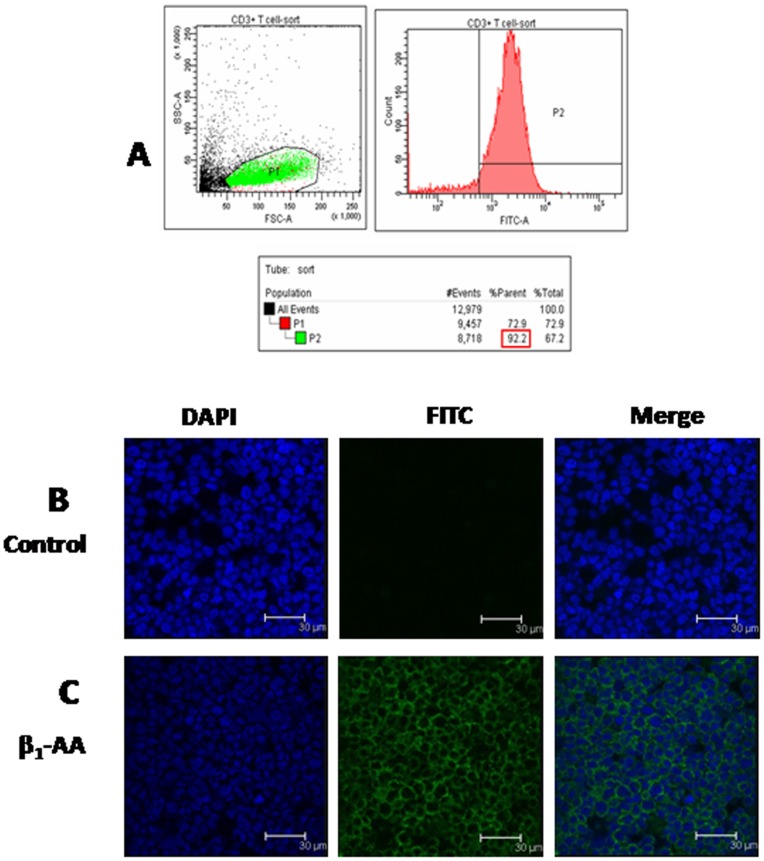
β_1_-AA from DCM patients bound to β_1_-ARs on the surface of CD3^+^T cells. A. After FACS, the purity of selected rat CD3^+^T lymphocytes by immunomagnetic separation was 92.2%. **B, C.** The binding of β_1_-AA (25 µg/ml) with the β_1_-ARs on the CD3^+^T cells was determined by confocal microscopy, and β_1_-AR was identified using an anti-β_1_-AR antibody (green). Nuclei were labeled with DAPI (blue). The negative control was performed by omitting primary antibodies during the incubation. Bar, 30 µm.

To determine the purity of the CD3^+^T cells preparation, multi-color flow cytometry was used. As illustrated in Fig. 2A, after sorting, the CD3^+^T cells represented 92.2% of the cell population. We next employed immunofluorescence staining to determine whether the IgG fraction isolated from the β_1_-AA-positive sera of DCM patients could bind to β_1_-ARs. We found that β_1_-AA (25 µg/ml) showed that the β_1_-AR staining was localized mainly to the membrane, while DAPI staining was only observed in the nucleus (Fig. 2C). Therefore, we concluded that the IgG fraction isolated from β_1_-AA-positive sera of DCM patients exhibited a pattern of β_1_-AR specific binding virtually identical to commercially available β_1_-AR-specific antibodies.

### β_1_-AA Increased the Beat Frequency of Cultured Cardiomyocytes

**Figure 3:**
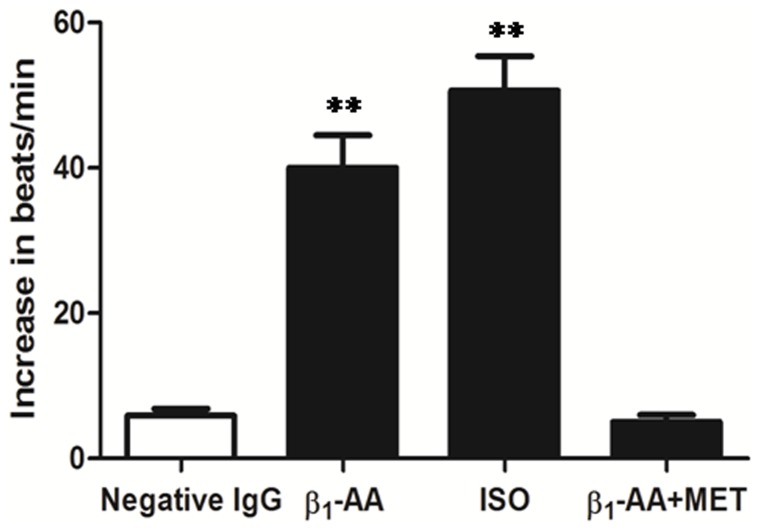
β_1_-AA from DCM patients increased the beat frequency of cultured cardiomyocytes. The bar graph shows the increase in beat frequency of isolated myocardial cells stimulated by β_1_-AA (25 µg/ml) or isoproterenol (0.1 µmol/l). ^**^ *p*<0.01 versus vehicle group. Data were presented as means ± SD of three independent experiments. ISO: isoproterenol, MET: metoprolol.

The IgG fractions (25 µg/ml) isolated from β_1_-AA-positive sera of DCM patients increased cardiomyocyte beat frequency, similar to the effects of the β_1_-adrenergic receptor agonist isoproterenol (0.1 µmol/l). This effect of β_1_-AA was abolished by the addition of the β_1_-adrenergic receptor antagonist metoprolol (1 µmol/l) (Fig. 3).

### β_1_-AA Promoted the Proliferation of T Lymphocytes

**Figure 4:**
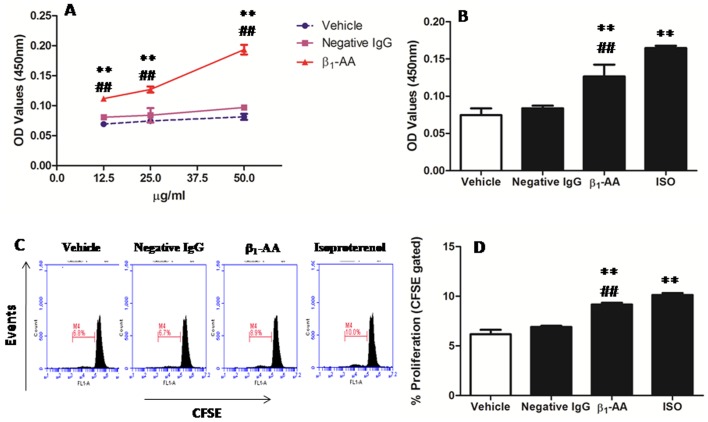
β_1_-AA from DCM patients significantly promoted the proliferation of CD3^+^T cells. **A.** β_1_-AA promoted CD3^+^T cell proliferation in a concentration-dependent manner. **B.** CD3^+^T cells (5×10^5^ cells/ml) were incubated for 48 h at 37°C and 5% CO_2_ in the presence of β_1_-AA (25 µg/ml) or isoproterenol (0.1 µmol/l). Cell proliferation was measured at 450 nm by CCK-8 uptake assay. ^**^ *p*<0.01 versus vehicle group; ^##^ *p*<0.01 versus negative IgG group. *n* = 9 per group. **C.** CD3^+^T cells were labeled with 4 µmol/l CFSE, and cell proliferation was measured by flow cytometry. Data shown here are representative of one of three different experiments with similar results. **D.** The bar graph shows the percentage of proliferating (CFSE^lo^) T cells among the total CD3^+^T cell population. *n* = 3, ^*^ *p*<0.05 versus vehicle group. ^**^ *p*<0.01 versus vehicle group; ^##^ *p*<0.01 versus Negative IgG group, ISO: isoproterenol.

We found that β_1_-AA enhanced T cell proliferation in a concentration-dependent manner (Fig. 4A). Therefore, we chose 25 µg/ml for further study because it is comparable to the concentration in the sera of heart failure patients [18]–[19]. Freshly isolated CD3^+^T cells were stimulated with anti-CD3/CD28 mAb in either the presence or absence of β_1_-AA for 48 h. As summarized in Fig. 4, the presence of β_1_-AA increased CD3^+^T cell proliferation (0.127±0.028 *vs.* 0.0745±0.016, *p*<0.05) (Fig. 4B). However, administration of β_1_-AA-negative IgG purified from 51 DCM patients did not detectably affect proliferation (0.084±0.0059 *vs.* 0.0745±0.016, *p*>0.05) (Fig. 4B). Similar results were obtained when we used the CFSE assay (Figs. 4C and 4D). Taken together, the results presented in Fig. 4 demonstrate that β_1_-AA present in DCM patients induced CD3^+^T cells proliferation.

### β_1_-AA Enhanced T Lymphocyte Proliferation through the β_1_-AR/cAMP/PKA Pathway

**Figure 5:**
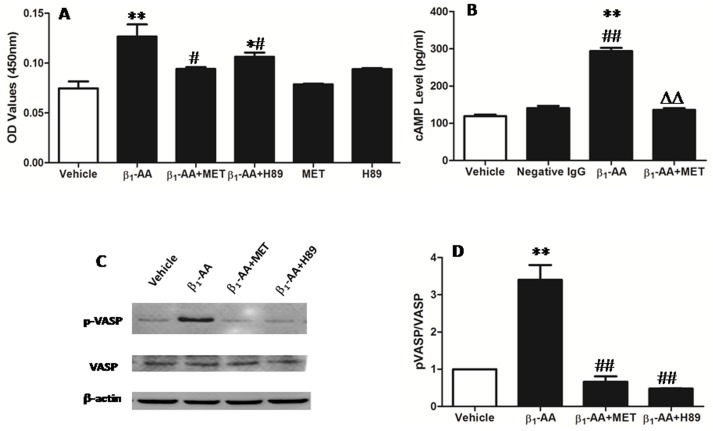
β_1_-AA-mediated T cells proliferation through the β_1_-AR/cAMP/PKA pathway. **A.** T cells were stimulated with the selective β_1_-AR antagonist metoprolol (1 µmol/l) and the selective PKA inhibitor H89 (1 µmol/l) for 1 h at 37°C in 5% CO_2_ before the addition of β_1_-AA (25 µg/ml). ^*^ *p*<0.05,^ **^ *p*<0.01 versus vehicle group; ^#^ *p*<0.05, ^##^ *p*<0.01 versus β_1_-AA group. *n* = 9 per group. **B.** The effect of β_1_-AA or isoproterenol on the production of cAMP (expressed as pg/ml) in T lymphocytes was examined by ELISA, ^**^ *p*<0.01 versus vehicle group, ^##^ *p*<0.01 versus negative IgG group, ^ΔΔ^ *p*<0.01 versus β_1_-AA group. Data were presented as means ± SD of 6 independent experiments. **C.** Immunoblot detection of phosphorylated VASP (p-VASP) and total VASP from CD3^+^T cells stimulated with β_1_-AA for 30 min. Images are representative of 3 independent experiments. **D.** The bar graph shows the ratio of p-VASP to total VASP. *n = *3, **p<0.01 versus vehicle group, ^ΔΔ^ *p*<0.01 versus β_1_-AA group, MET: metoprolol.

The most common signaling mechanism initiated by β_1_-AR stimulation is the β_1_-AR/cAMP/PKA pathway [18], [20]. To determine whether β_1_-AA-stimulated T cell proliferation resulted from the triggering of this pathway, the selective β_1_-AR antagonist metoprolol (1 µmol/l) and the PKA inhibitor H89 (1 µmol/l) were used to block the pathway before β_1_-AA administration, and then the activity of PKA was determined. The results demonstrated that T cell proliferation mediated by β_1_-AA was partially inhibited by metoprolol (0.094±0.0044 *vs.* β_1_-AA group 0.127±0.028, *p*<0.01) (Fig. 5A) and H89 (0.106±0.0097 *vs.* β_1_-AA group 0.127±0.028, *p*<0.05; 0.106±0.0097 *vs.* vehicle group 0.107±0.006, *p*>0.05) (Fig. 5A). Additionally, we determined the accumulation of intracellular cAMP in T lymphocytes stimulated with β_1_-AA (25 µg/ml). As summarized in Fig. 5B, basal levels of cAMP (119±9.63 pg/ml) were detected in T lymphocytes stimulated with anti-CD3/CD28 mAb alone. β_1_-AA significantly enhanced accumulation of intracellular cAMP levels (294±19.4 pg/ml, *p*<0.01), whereas metoprolol (1 µmol/l) antagonized the β_1_-AA-induced accumulation of cAMP (Fig. 5B). In the immunoblot analysis, β_1_-AA stimulated VASP phosphorylation, which was inhibited by metoprolol and H89. However, it had no effect on total VASP (Fig. 5C, 5D). Collectively, these results suggest that the β_1_-AR/cAMP/PKA pathway was involved in T cell proliferation stimulated by β_1_-AA.

### Involvement of p38 MAPK in β_1_-AA-mediated T Lymphocyte Proliferation

**Figure 6:**
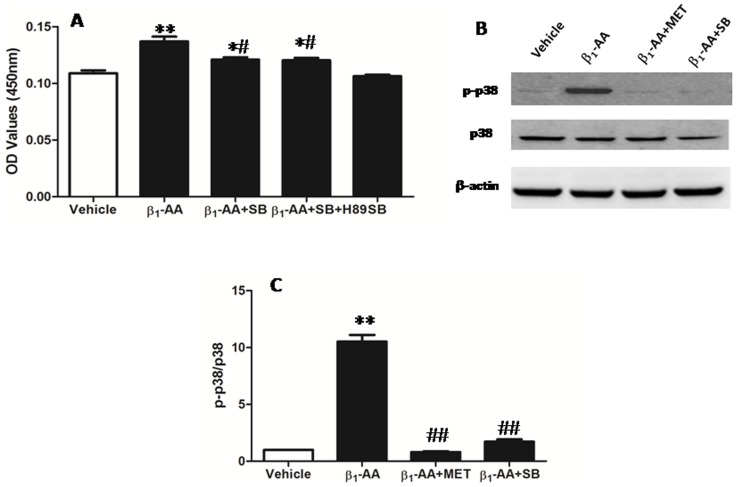
β_1_-AA-mediated T cell proliferation mediated by activation of p38 MAPK. **A.** T cells were pretreated with the selective PKA inhibitor H89 (1 µmol/l) and the selective p38 MAPK inhibitor SB203580 (1 µmol/l) for 1 h at 37°C in 5% CO_2_ before stimulation by β_1_-AA (25 µg/ml). *p<0.05, **p<0.01 versus vehicle group; ^#^ *p*<0.05 versus β_1_-AA group. *n* = 9 per group. **B.** Immunoblot detection of phosphorylated p38 MAPK (p-p38 MAPK) and total p38 MAPK from CD3^+^T cells stimulated with β_1_-AA for 30 min. Images are representative of three independent experiments. **C. **The bar graph shows the ratio of p-p38 MAPK to total p38 MAPK. *n* = 3, **p<0.01 versus Vehicle group, ^ΔΔ^ *p*<0.01 versus β_1_-AA group, SB: SB203580.

In immune cells, p38 MAPK plays a role in regulating the production of mature T cells [21]–[22]. To investigate the role of activation of p38 MAPK in β_1_-AA-stimulated T cell proliferation, the selective p38 MAPK antagonist SB203580 (1 µmol/l) was used to block the pathway before stimulation with β_1_-AA (25 µg/ml), and then the activity of p38 MAPK was determined. As depicted in Fig. 6A, T cell proliferation stimulated by β_1_-AA was partially inhibited by SB203580 (0.121±0.00415 *vs.* β_1_-AA group 0.137±0.0086, *p*<0.05; 0.121±0.00415 *vs.* vehicle group 0.109±0.0052, *p*<0.05). Although H89 and SB203580 were used together before β_1_-AA administration, the proliferation of T cells induced by β_1_-AA was still partially blocked (0.12±0.0043 *vs.* β_1_-AA group 0.137±0.0086, *p*<0.05; 0.12±0.0043 *vs.* vehicle group 0.109±0.0052, *p*<0.05) (Fig. 6A). In immunoblot analysis, β_1_-AA treatment of anti-CD3/CD28-mAb-activated T cells resulted in increased p38 MAPK phosphorylation, while total p38 MAPK remained unchanged (Fig. 6B, 6C). These data demonstrate a role for p38 MAPK activation in T cell proliferation mediated by β_1_-AA. Taken together, both the β_1_-AR/cAMP/PKA pathway and p38 MAPK activation were involved in the production of mature T cells.

### β_1_-AA Inhibited IFN-γ Secretion, but Promoted IL-4 Production

**Figure 7:**
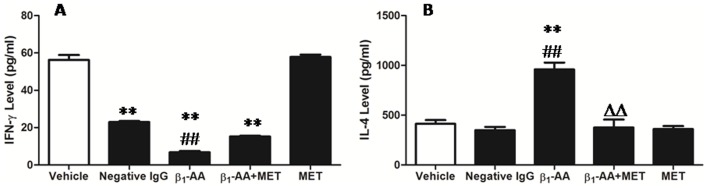
β_1_-AA inhibited IFN-γ secretion and promoted IL-4 production in T cells. **A.** T cells (5 × 10^5^ cells/ml) were pretreated with 1 µmol/l metoprolol in the presence of β_1_-AA (25 µg/ml) for 48 h, and then IFN-γ levels were analyzed by ELISA. ^**^ *p*<0.01 versus vehicle group, ^##^ *p*<0.01 versus negative IgG group, *n* = 9/group **B.** The effect of β_1_-AA (25 µg/ml) on IL-4 levels was examined by ELISA,^ **^ *p*<0.01 versus vehicle group, ^##^ *p*<0.01 versus negative IgG group, ^ΔΔ^ *p*<0.01 versus β_1_-AA group, *n* = 9 per group. Data are presented as means ± SD of 6 independent experiments.

As illustrated in Fig. 7A, the addition of IgG isolated from β_1_-AA-positive sera of DCM patients caused a reduction in IFN-γ production (6.788±1.46 pg/ml *vs.* vehicle group 56.22±2.29 pg/ml, *p*<0.01; 6.788±1.46 pg/ml *vs.* Negative IgG group 22.96±0.905 pg/ml, *p*<0.01) (Fig. 7A). The effect was completely blocked by the addition of the selective β_1_-AR antagonist metoprolol (1 µmol/l) (*p*>0.05) (Fig. 7A). We next examined the effects of β_1_-AA on the production of IL-4 in T cells. The results suggest that β_1_-AA promoted the secretion of IL-4 (959.37±61.79 pg/ml *vs.* 413.19±32.495 pg/ml, *p*<0.01) (Fig. 7B), while the increase in IL-4 was antagonized by metoprolol (1 µmol/l) (*p*>0.05). Collectively, these results suggest that β_1_-AA regulated the secretion of T cells.

## Discussion

The concentration of circulating autoantibodies directed against the second extracellular loop of β_1_-AR is known to be increased in patients with heart failure [17] when compared with healthy subjects. Our present results confirm these findings, and we report here that autoantibodies directed against the second extracellular loop of β_1_-AR were present in 46.3% of sera collected from 95 patients with heart failure due to DCM, which was significantly higher than in the samples from 95 healthy subjects. Moreover, we noted that their overall prevalence was 8.42% in healthy subjects, which is clearly more than has been reported to date [19], [20], [23]. We believe that these differences are essentially due to different methods used to detect the autoantibodies as well as the discriminant criteria. In the present study, the P/N ratio was used to represent the positive rate of β_1_-AA detection, which can effectively reduce the risk for false negative results [6]. Furthermore, during the myocardial remodeling process in rats, the generation of β_1_-AA showed a characteristic self-growth and time-course decline, and the existence of β_1_-AA in rats lasted for a short period, with the titers gradually tapering after about two to three months [2]. Additionally, in concert with our animal experiments, it was reported, clinically, that autoantibodies against the β_1_-AR existing in the sera of DCM patients also present a time-course decrease and cardiac autoantibodies in patients with DCM become undetectable with disease progression [24]–[25]. Therefore, the presence of β_1_-AA in the sera of DCM patients showed a time-course decrease, and the different pathological processes of heart failure patients could lead to the changes of incidence of β_1_-AA. Moreover, using the immunofluorescence and radioligand-binding techniques, we could demonstrate that β_1_-AA isolated from DCM patients recognized β_1_-ARs expressed either on CD3^+^T cells or on H9c2 rat cardiomyoblast cells transiently transfected with β_1_-AR (Fig. S1C, Fig. 2), whereas after β_2_-ARs expressed on CD3^+^T cells were blocked by the specific β_2_-AR antagonist ICI118551, CD3^+^T cells can still be stained (Fig. S1B). Additionally, in order to enhance specificity, the monoclonal antibody, which was obtained by immunizing Balb/C mice with free peptide H26R corresponding to the second extracellular loop of the human β_1_-AR, and had the same biological effect with β_1_-AAIgGs isolated from heart failure patients (Fig. S3), was used to serve as positive control (Fig. S1A). These experiments revealed that β_1_-AA isolated from DCM patients can bind to β_1_-ARs on CD3^+^T cells, and did not cross-react with the very closely related β_2_-AR expressed on CD3^+^T cells. All of these antibodies were directed against the second extracellular domain, which is known to affect ligand binding [26] and may induce immune responses [11]. They all increased the beat frequency and cAMP level of cultured cardiomyocytes (Fig. S4), in the same way as the effect of IgG fractions isolated from individual β_1_-AA-positive sera of DCM patient (Fig. S5). Our results indicated that β_1_-AA may be one of the abnormal immune phenomena in heart failure and suggests their involvement in the pathophysiology of essential heart failure.

Recent clinical reports showed that the selective β_1_-AR antagonist, metoprolol, decreased the frequency and the geometric mean titer of β_1_-AA [23], [27]. Moreover, Wallukat [28] demonstrated that the β_1_-adrenergic receptor antagonists were able to block the effect of the antibodies and displace the anti–β_1_-adrenergic receptor antibodies from their binding sites on the receptor, leading to a decrease of β_1_-AA titers in patients with heart failure. Therefore, the DCM patients involved in the current study were required to stop treatment with β_1_-AR antagonists for one week.

T lymphocytes recognize antigens, help or suppress B cells to produce antibodies, and secrete cytokines [29]. The activation and proliferation of T lymphocytes are required for these functions. Therefore, the state of immune function is usually reflected by the proliferation of T lymphocytes. In the present study, in order to exclude the interference of other cells, FACS was used to separate CD3^+^T lymphocytes to specifically determine how they are affected by β_1_-AA. We found that β_1_-AA isolated from DCM patients had no effect on the numbers of resting CD3^+^T cells (Fig. S6A), but it can enhance the proliferation of anti-rat CD3/CD28 mAbs-induced CD3^+^T cells. Therefore, in this study, the CD3^+^T cells were stimulated with anti-rat CD3/CD28 mAbs first, and then the effect of β_1_-AA on activated CD3^+^T cells was analyzed. Furthermore, we have isolated CD3^+^T cells from β_1_-AA-positive, -negative DCM patients and healthy control subjects. Additionally, the CD3^+^T cells were stimulated with anti-human CD3/CD28 mAbs first, and then the role of β_1_-AA in the proliferation of activated CD3^+^T cells was observed. We found that β_1_-AA from DCM patients significantly enhanced the proliferation of CD3^+^T cells isolated from β_1_-AA-positive and -negative DCM patients as well as healthy control subjects (Fig. S6B, C, D).

According to published paper [14], the patients with DCM also develop functionally active antibodies against the first extracellular loop of the β_1_-AR (β_1_-AR-EC_I_). Therefore, to detect whether there were anti-β_1_-AR-EC_I_-antibodies in DCM patients involved in this study, we added peptide corresponding to the sequence of the 1st (β_1_-AR-EC_I_) as negative control, and found that the supernatant produced by incubating β_1_-AA-positive IgG with β_1_-AR-EC_I_ still promoted CD3^+^T lymphocytes proliferation (Fig. S7A). However, the supernatant produced by incubating β_1_-AA-positive IgG with peptide corresponding to the sequence of the 2nd (β_1_-AR-EC_II_) had no effect on CD3^+^T lymphocytes (Fig. S7B). These results suggest that β_1_-AA-positive IgG isolated from DCM patients promoted the proliferation of CD3^+^T lymphocytes by binding to β_1_-AR-EC_II_.

The most common signaling mechanism initiated by β_1_-AR stimulation is the β_1_-AR/cAMP/PKA pathway [18], [20]. In order to explore whether β_1_-AA promoted T lymphocyte proliferation through this pathway, the β_1_-AR selective antagonist metoprolol was added to T lymphocytes before β_1_-AA administration. As a result, the proliferative effect could be blocked, suggesting that β_1_-ARs on the surface of CD3^+^T cells could be activated by the β_1_-AA from DCM patients. Furthermore, we also found that each β_1_-AA sample increased mitogen-stimulated cAMP production in a receptor-mediated fashion.

Previous studies have demonstrated the PKA-dependent effects in immune cells by either assessing agonist-stimulated PKA activity through *in vitro* assays or demonstrating the actions of pharmacologic PKA inhibitors and activators [30]–[31]. Here we used a more direct approach in which we analyzed PKA activity by assaying for the phosphorylation of VASP at Ser157, which is mediated directly and selectively by PKA [32]–[33]. We found that β_1_-AR activation by β_1_-AA rapidly led to VASP phosphorylation at Ser157. The selective β_1_-AR antagonist metoprolol decreased the level of VASP phosphorylation stimulated by β_1_-AA. In addition, inhibition of PKA by compound H89 abrogated β_1_-AA-induced phosphorylation of VASP at Ser157. However, β_1_-AA had no effect on total VASP. Taken together, all of these results strongly implicate the β_1_-AR/cAMP/PKA pathway as the principal signaling system modulating the β_1_-AA-induced phosphorylation of VASP at Ser157.

In T cells, one physiological effect of p38 MAPK activity is the regulation of cell growth and cell death, which are especially important in the thymus during T cell development [34]. Dysregulation of p38 MAPK can result in negative selection-induced cell death and the subsequent absence of T cell populations in the peripheral immune system [35]. Our present results suggest that the proliferation of T cells induced by β_1_-AA was partially blocked by the p38 MAPK-selective inhibitor SB203580. Moreover, immunoblot assays revealed an apparent increase in phosphorylation of p38 MAPK following treatments with β_1_-AA, in the absence of an effect on total p38 MAPK levels. These results indicated that activation of p38 MAPK was correlated with the production of mature T cells. Furthermore, when H89 and SB203580 were used together before stimulation with β_1_-AA, the proliferation of T cells was also partially inhibited. These results suggest that both β_1_-AR/cAMP/PKA and p38 MAPK pathways contributed to β_1_-AA-mediated proliferation of T cells. However, other pathways may also participate in this process. The study by Antonio et al. [18] showed that the effect of β_1_-AA on cardiomyocytes could be blocked by tyrosine kinase inhibitor PP2. Other studies have also reported that the role of β_1_-AR could be mediated by PI3-kinase, PKC, or PKA in the trigger phase of ischemic preconditioning [36]. Based on the studies mentioned, further researches are necessary to investigate the other possible pathways stimulated by β_1_-AA.

The main form of T cell activation is to secrete cytokines. Therefore, in the current study, the levels of IFN-γ and IL-4, the characteristic cytokines secreted by T cells, were chosen to detect the effect of β_1_-AA on T lymphocytes secretion. IFN-γ is a major proinflammatory effector and regulatory cytokine produced by activated T lymphocytes, which can inhibit humoral immunity by suppressing the production of Th2 cells, but promotes cell-mediated immunity [37]. Our results suggest that both β_1_-AA-positive and -negative IgGs might inhibit the secretion of IFN-γ, though the effect of β_1_-AA-positive IgGs was more pronounced than that of β_1_-AA-negative IgGs. However, the IgGs isolated from healthy subjects did not enhance IFN-γ secretion (Fig. S8A). Moreover, in order to analyze whether the reduction of IFN-γ production caused by the β_1_-AA-negative IgG preparation of DCM patients might have an effect of anti-β_1_-AR-EC_I_-antibodies, we added peptide corresponding to the sequence of the 1st extracellular loop of the receptor (β_1_-AR-EC_I_) as a negative control. We found unexpectedly that the supernatant produced by incubating β_1_-AA-negative IgGs with β_1_-AR-EC_I_ also decreased IFN-γ production, however, compared with β_1_-AA-negative IgGs, the inhibition of IFN-γ level was alleviated (Fig. S9). These results suggest that there may be anti-β_1_-AR-EC_I_-antibodies in β_1_-AA-negative DCM patients, which inhibited IFN-γ production. Besides that, heart failure itself may be a risk factor in inhibiting cell-mediated immunity, and the effect may be magnified due to the presence of β_1_-AA. However, the mechanism for the β_1_-AA-mediated decrease in IFN-γ production is unknown, and further investigations are needed to explain this phenomenon.

IL-4 is the characteristic cytokine secreted by Th2 cells, which decreases the production of Th1 cells and promotes humoral immunity [38]–[39]. It has been reported that the β-AR agonist isoproterenol may activate Th2 cells and promote IL-4 production mainly via binding to β_2_-ARs expressed on T lymphocytes [40]. In the current study, we found that β_1_-AA from DCM patients can enhance IL-4 release through combining with β_1_-ARs also expressed on T lymphocytes, but the IgGs isolated from healthy subjects did not enhance IL-4 secretion (Fig. S8B). These results suggest that although β_1_-AA mediates β_1_-AR agonist-like actions; it is different from the β_1_-AR agonist isoproterenol and might activate Th2 cells through the β_1_-AR pathway. Additionally, β_1_-AA serves as a kind of antibody that is produced by B cells and Th2 cells, and the present study shows that β_1_-AA may enhance humoral immunity, while inhibiting cell-mediated immunity. This suggests that there is positive feedback between β_1_-AA and Th2 cells. However, further studies are required to support this conclusion.

We observed here the direct effect of IgG fractions from β_1_-AA-positive sera on CD3^+^T lymphocytes isolated from patients with heart failure due to DCM, and demonstrated that T lymphocyte, in addition to cardiomyocyte, may also be one of the important targets of β_1_-AA isolated from DCM patients. The vicious circle between the immune system and β_1_-AA may exacerbate autoantibody-positive diseases.

Nonetheless, our work leaves some unanswered questions and paths for future work. The β_1_-AA used in this study was not specific for the second extracellular loop of β_1_-AR, and some nonspecific IgGs were involved. Additionally, because of clinical and ethical reasons, DCM patients involved in the current study only stopped β-blocker therapy for one week, which can not completely preclude effects of β-blocker on β_1_-AA synthesis. Therefore, further studies using monoclonal antibodies specific for the second extracellular loop of β_1_-AR would be carried out to yield more conclusive results. Moreover, whether there are any differences in T lymphocytes in β_1_-AA positive and negative patients has not been elucidated. In addition, in the present study, ELISA and beating rate of isolated neonatal cardiomyocytes were employed to detect the titer and function of β_1_-AA in the patients with DCM. However, recent research reported that a novel molecular and/or fluorescence-based diagnostic method of detecting β_1_-AA in patients with heart failure was proved to be fast and highly sensitive [41]. Therefore, further investigations using new diagnostic methods would be conducted to provide functional and conclusive diagnostic data.

## Supporting Information

Figure S1 **Colocalization experiments. A.** Anti-β_1_-AR monoclonal antibody was used as a positive control. **B.** CD3^+^T cells were pretreated with ICI118551 for 1 h in the presence of β_1_-AA, and then the binding of β_1_-AA with the β_2_-ARs on CD3^+^T cells was determined by confocal microscopy respectively. **C.** Colocalization experiments with H9c2 cells transiently expressing β_1_-AR.
(TIF)

Figure S2 **Inhibitory effect of β_1_-AA from DCM patients with different concentrations on [^125^I]-PIN binding to β_1_-AR.** Results are expressed as percentage of binding in the absence of β_1_-AA.
(TIF)

Figure S3 **Anti-β_1_-AR monoclonal antibody has been synthesized successfully. A.** The level of β_1_-AA in the supernatant of hybridoma cell was detected using ELISA. **p < 0.01 *vs.* Vehicle group; n = 3/group. **B.** Western blot method was used to analyze the combination between anti-β_1_-AR monoclonal antibody and β_1_-AR on the surface of H9C_2_ cell. n = 3/group. Supernatant group: the supernatant of hybridoma cell, Positive control antibody group: commercial anti-β_1_-AR polyclonal antibody. **C.** Radioligand-binding experiment was employed to investigate the co-localization of anti-β_1_-AR monoclonal antibody to the β_1_-ARs on the surface of H9C_2_ cell. **D.** Anti-β_1_-AR monoclonal antibody increased the beat frequency of cultured cardiomyocytes. The bar graph shows the increase in beat frequency of isolated myocardial cells stimulated by anti-β_1_-AR monoclonal antibody (25 µg/ml) or β_1_-AA isolated from DCM patients (25 µg/ml). Data were presented as means ± SD of three independent experiments.
(TIF)

Figure S4 Increases in basal cAMP levels in cultured neonatal rat cardiomyocytes expressing β_1_-AR upon incubation with β_1_-AA. ** *p*<0.01 versus vehicle group. *n* = 6 per group. Data are presented as means ± SD of 3 independent experiments.
(TIF)

Figure S5 **Functional assays with β_1_-AA purified from individual DCM patient. A.** β_1_-AA from individual DCM patient increased the beat frequency of cultured cardiomyocytes. **B.** Increases in basal cAMP levels in cultured neonatal rat cardiomyocytes incubation with β_1_-AA from individual DCM patient. ** *p*<0.01 versus vehicle group. *n* = 6 per group. Data are presented as means ± SD of 3 independent experiments.
(TIF)

Figure S6 **The effects of β_1_-AA on CD3^+^T cells proliferation. A**. β_1_-AA had no effect on resting rat CD3^+^T cells. **B, C, D**. β_1_-AA enhanced the proliferation of activated CD3^+^T cells isolated from β_1_-AA-positive/−negative DCM patients and healthy subjects, respectively. **p<0.01 versus vehicle group; ^##^ *p*<0.01 versus negative IgG group. *n* = 6 per group. Data are presented as means ± SD of 3 independent experiments.
(TIF)

Figure S7 **The proliferation of CD3^+^T lymphocytes induced by β_1_-AA was blocked by β_1_-AR-EC_II_.** The supernatant produced by incubating β_1_-AA with β_1_-AR-EC_I_ promoted CD3^+^T lymphocytes proliferation. The proliferation of CD3^+^T lymphocytes induced by β_1_-AA was blocked by β_1_-AR-EC_II_. **p < 0.01, *p < 0.05 versus. vehicle group. *n* = 6 per group. Data are presented as means ± SD of 3 independent experiments.
(TIF)

Figure S8 **The IgGs isolated from healthy subjects revealed no effect on the levels of IFN-γ and IL-4 in CD3^+^T cells.** *n* = 6 per group. Data are presented as means ± SD of 3 independent experiments.
(TIF)

Figure S9 **The reduce of IFN-γ induced by β_1_-AA-negative IgG was partially blocked by β_1_-AR-EC_I_.** β_1_-AA-negative IgG (25 µg/ml) and the peptide corresponding to the sequence of the first extracellular loop of the human β_1_-AR (β_1_-AR-EC_I_, 1 µmol/l) co-incubated for 1 h at 37°C, supernatants were then collected to treat CD3^+^T lymphocytes, and finally IFN-γ level was analyzed by ELISA. **p < 0.01, *p < 0.05 versus. vehicle group; ^ΔΔ^ *p*<0.01 versus negative IgG group. *n* = 6 per group. Data are presented as means ± SD of 3 independent experiments.
(TIF)
