# Supplementary figures and images for: β1-Adrenoceptor Autoantibodies from DCM Patients Enhance the Proliferation of T Lymphocytes through the β1-AR/cAMP/PKA and p38 MAPK Pathways
Source: PLoS One. 2012 Dec 31;7(12):e52911. doi: 10.1371/journal.pone.0052911 (PMC3534136; doi:10.1371/journal.pone.0052911)

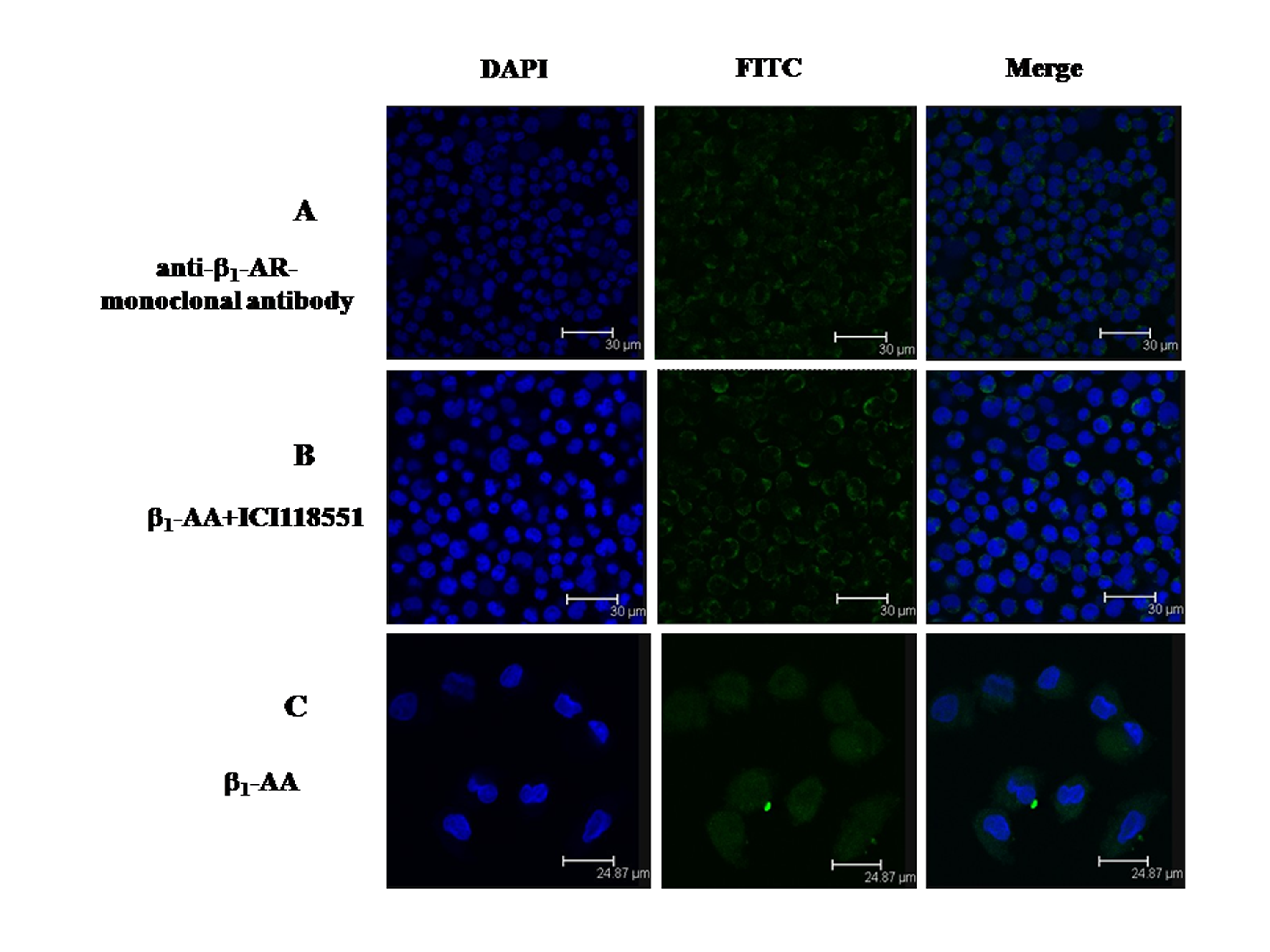

Supplement: Figure S1 — Colocalization experiments. A. Anti-β1-AR monoclonal antibody was used as a positive control. B. CD3+T cells were pretreated with ICI118551 for 1 h in the presence of β1-AA, and then the binding of β1-AA with the β2-ARs on CD3+T cells was determined by confocal microscopy respectively. C. Colocalization experiments with H9c2 cells transiently expressing β1-AR. (TIF) [file pone.0052911.s001.tif]

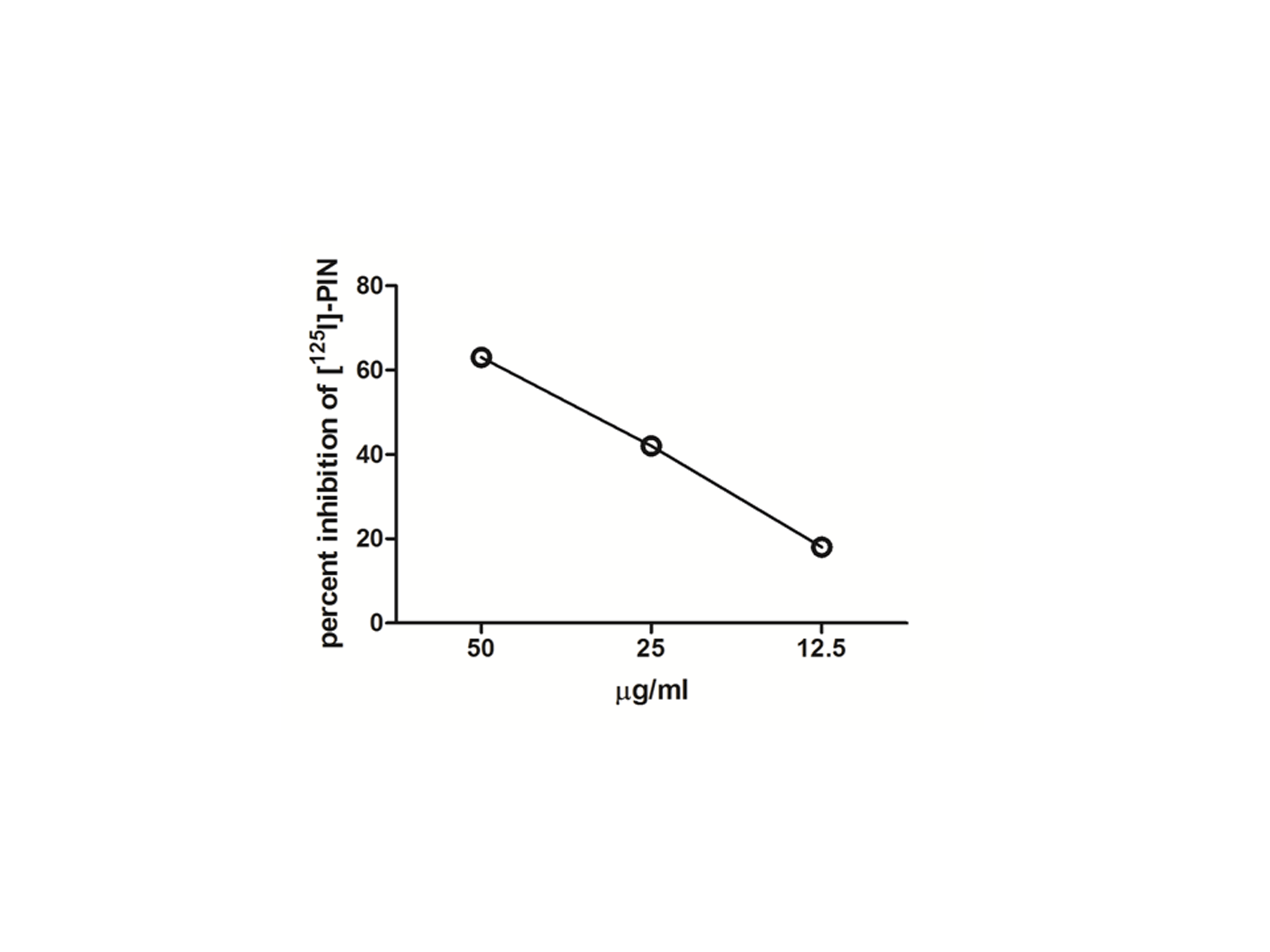

Supplement: Figure S2 — Inhibitory effect of β1-AA from DCM patients with different concentrations on [125I]-PIN binding to β1-AR. Results are expressed as percentage of binding in the absence of β1-AA. (TIF) [file pone.0052911.s002.tif]

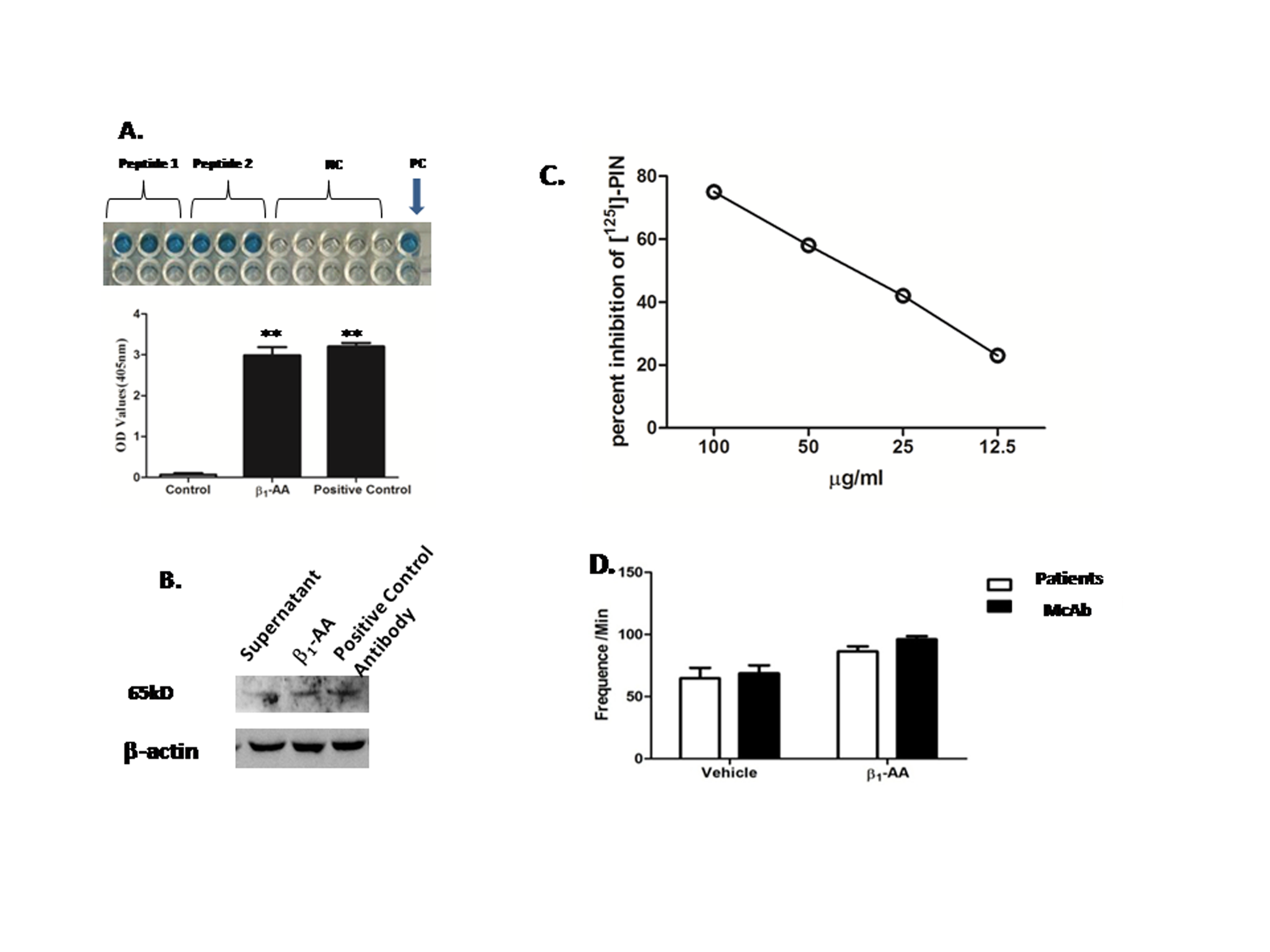

Supplement: Figure S3 — Anti-β1-AR monoclonal antibody has been synthesized successfully. A. The level of β1-AA in the supernatant of hybridoma cell was detected using ELISA. **p < 0.01 vs. Vehicle group; n = 3/group. B. Western blot method was used to analyze the combination between anti-β1-AR monoclonal antibody and β1-AR on the surface of H9C2 cell. n = 3/group. Supernatant group: the supernatant of hybridoma cell, Positive control antibody group: commercial anti-β1-AR polyclonal antibody. C. Radioligand-binding experiment was employed to investigate the co-localization of anti-β1-AR monoclonal antibody to the β1-ARs on the surface of H9C2 cell. D. Anti-β1-AR monoclonal antibody increased the beat frequency of cultured cardiomyocytes. The bar graph shows the increase in beat frequency of isolated myocardial cells stimulated by anti-β1-AR monoclonal antibody (25 µg/ml) or β1-AA isolated from DCM patients (25 µg/ml). Data were presented as means ± SD of three independent experiments. (TIF) [file pone.0052911.s003.tif]

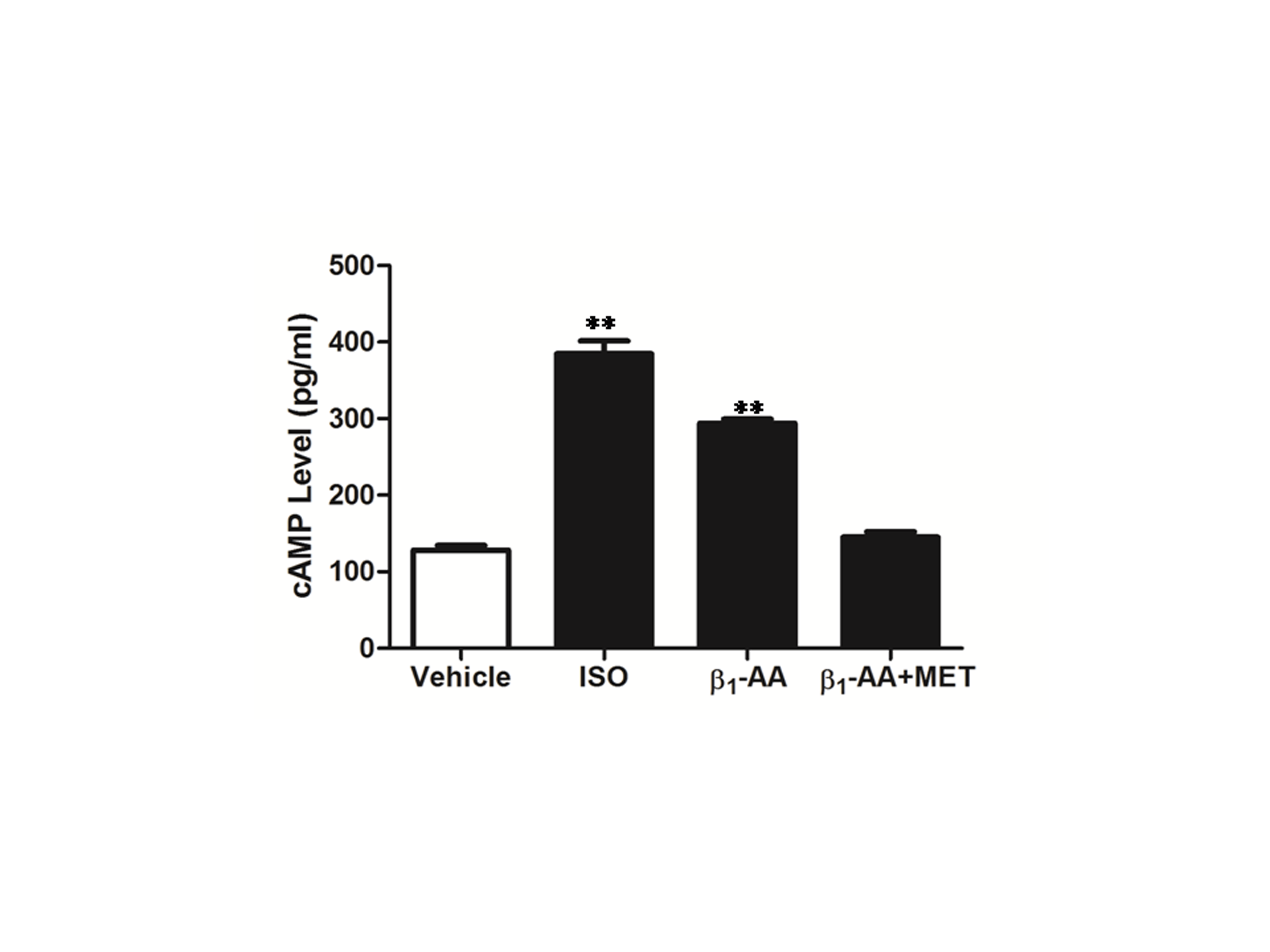

Supplement: Figure S4 — Increases in basal cAMP levels in cultured neonatal rat cardiomyocytes expressing β1-AR upon incubation with β1-AA. ** p<0.01 versus vehicle group. n = 6 per group. Data are presented as means ± SD of 3 independent experiments. (TIF) [file pone.0052911.s004.tif]

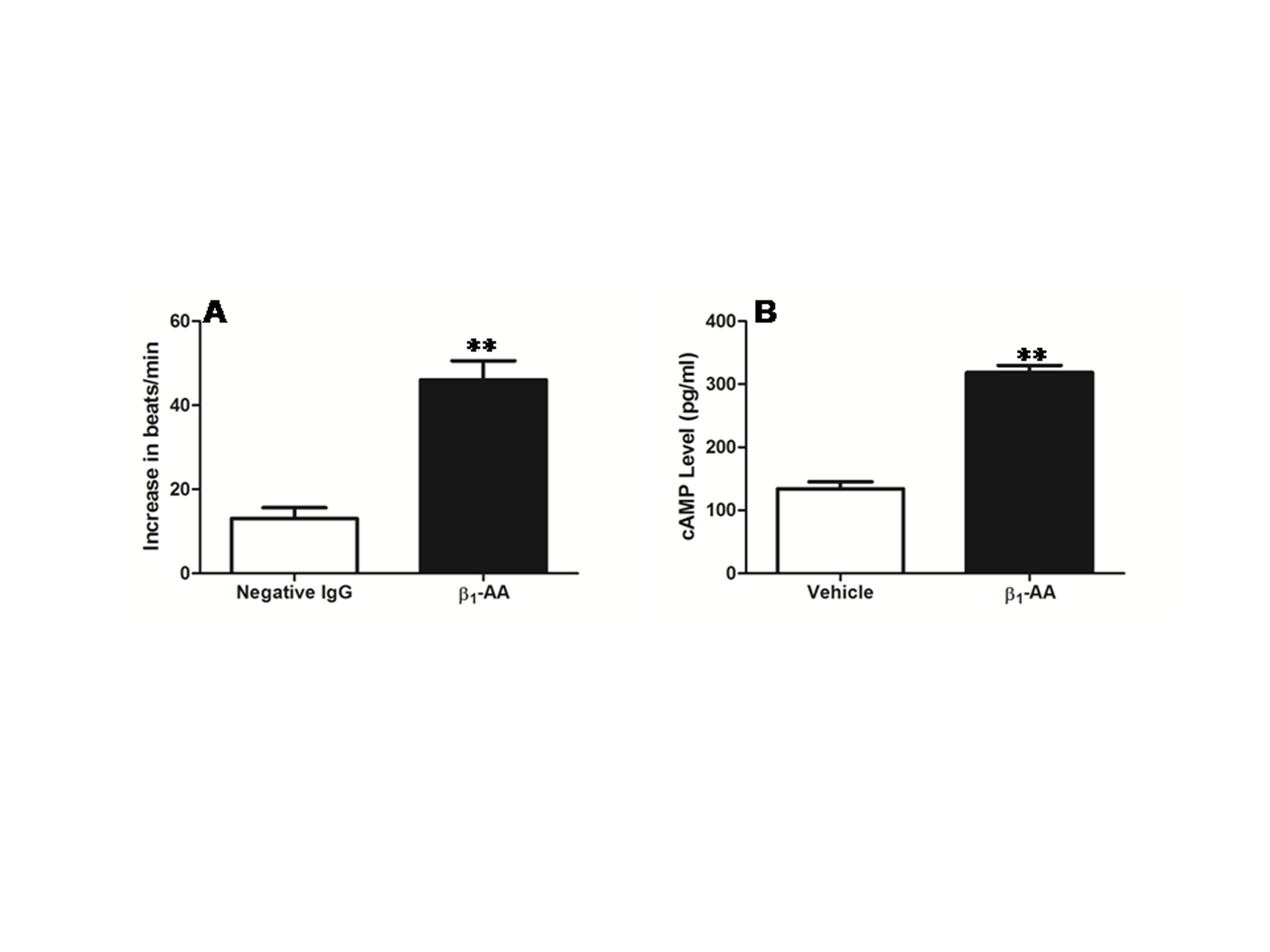

Supplement: Figure S5 — Functional assays with β1-AA purified from individual DCM patient. A. β1-AA from individual DCM patient increased the beat frequency of cultured cardiomyocytes. B. Increases in basal cAMP levels in cultured neonatal rat cardiomyocytes incubation with β1-AA from individual DCM patient. ** p<0.01 versus vehicle group. n = 6 per group. Data are presented as means ± SD of 3 independent experiments. (TIF) [file pone.0052911.s005.tif]

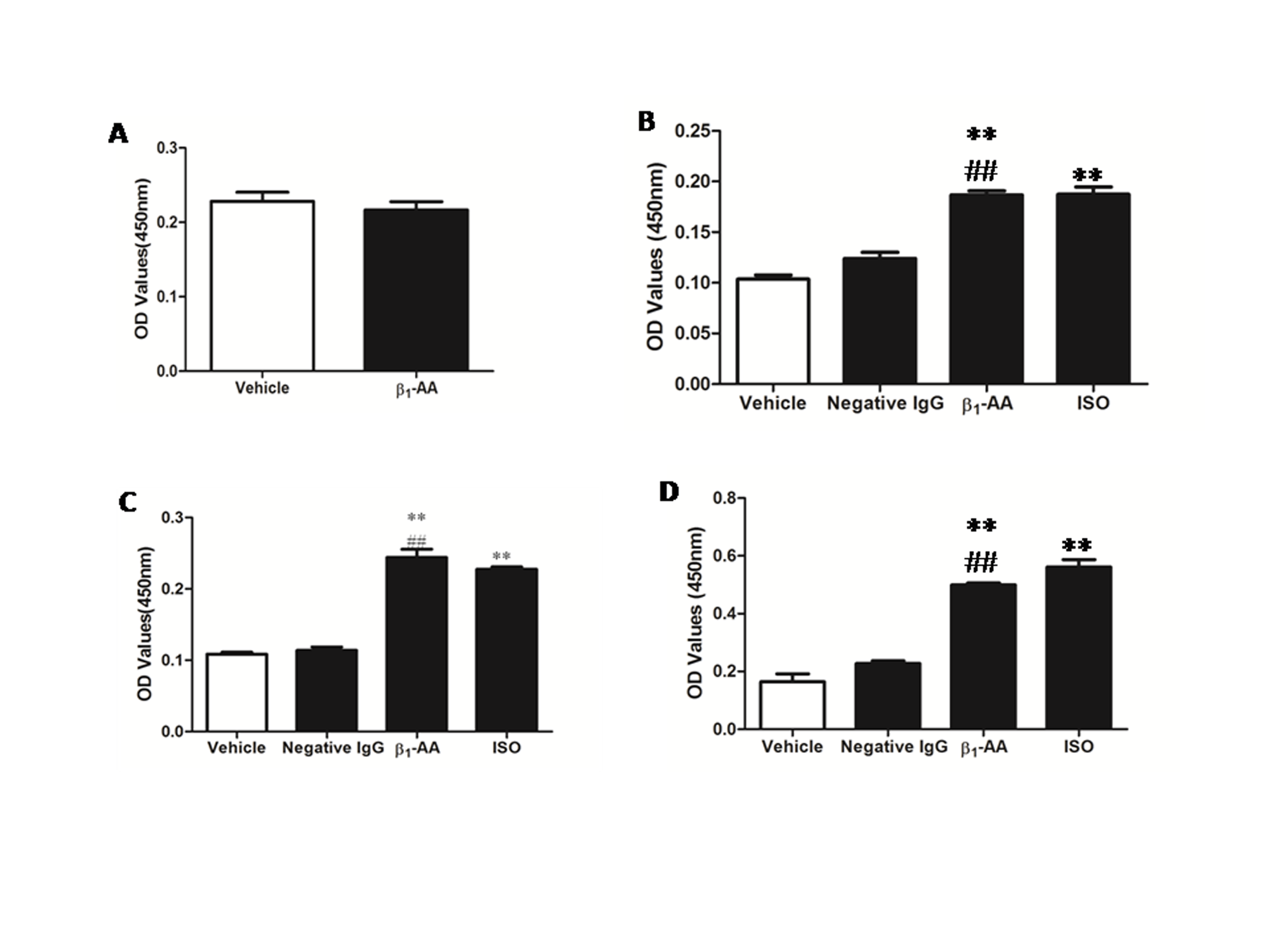

Supplement: Figure S6 — The effects of β1-AA on CD3+T cells proliferation. A. β1-AA had no effect on resting rat CD3+T cells. B, C, D. β1-AA enhanced the proliferation of activated CD3+T cells isolated from β1-AA-positive/−negative DCM patients and healthy subjects, respectively. **p<0.01 versus vehicle group; ## p<0.01 versus negative IgG group. n = 6 per group. Data are presented as means ± SD of 3 independent experiments. (TIF) [file pone.0052911.s006.tif]

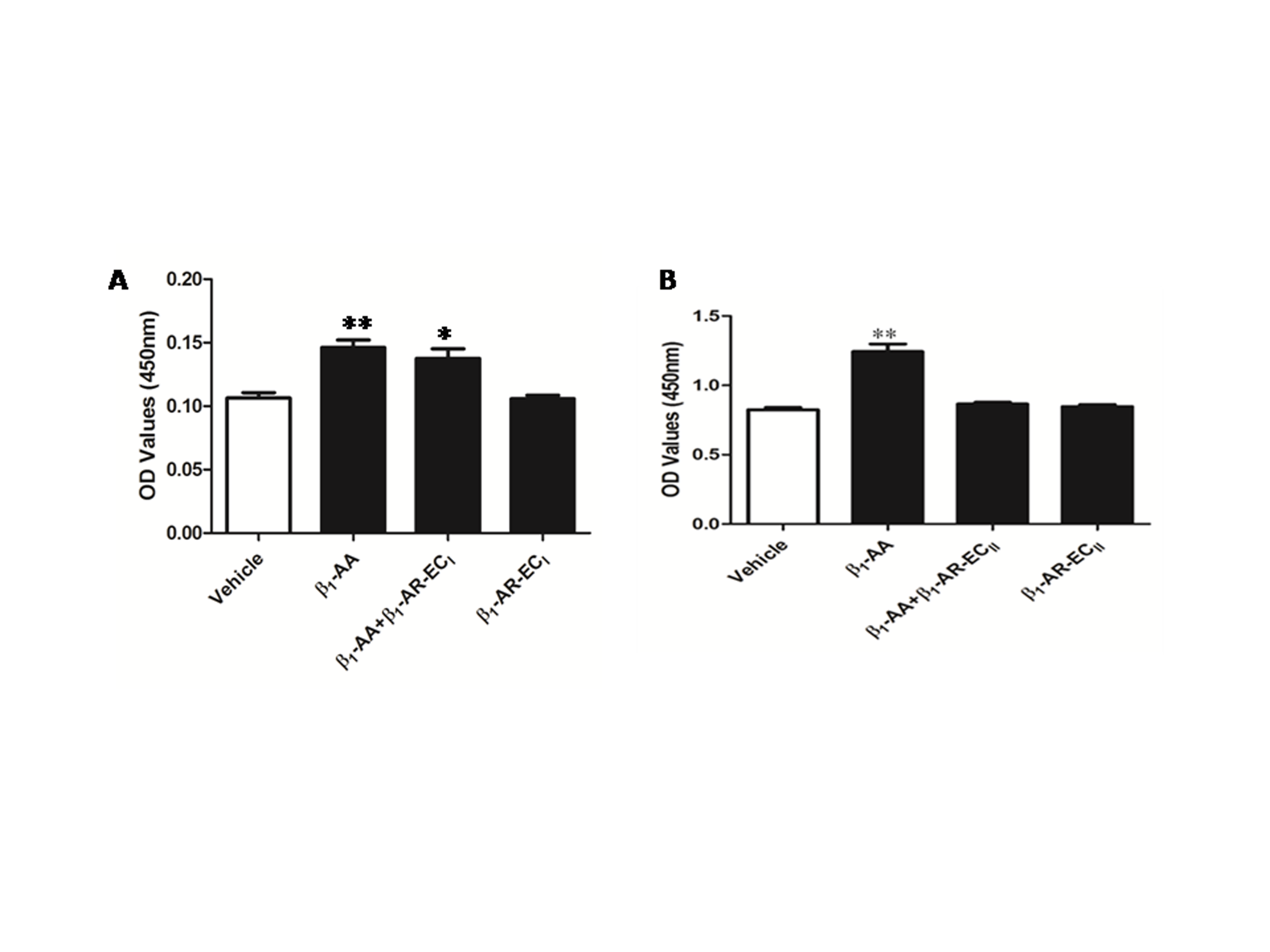

Supplement: Figure S7 — The proliferation of CD3+T lymphocytes induced by β1-AA was blocked by β1-AR-ECII. The supernatant produced by incubating β1-AA with β1-AR-ECI promoted CD3+T lymphocytes proliferation. The proliferation of CD3+T lymphocytes induced by β1-AA was blocked by β1-AR-ECII. **p < 0.01, *p < 0.05 versus. vehicle group. n = 6 per group. Data are presented as means ± SD of 3 independent experiments. (TIF) [file pone.0052911.s007.tif]

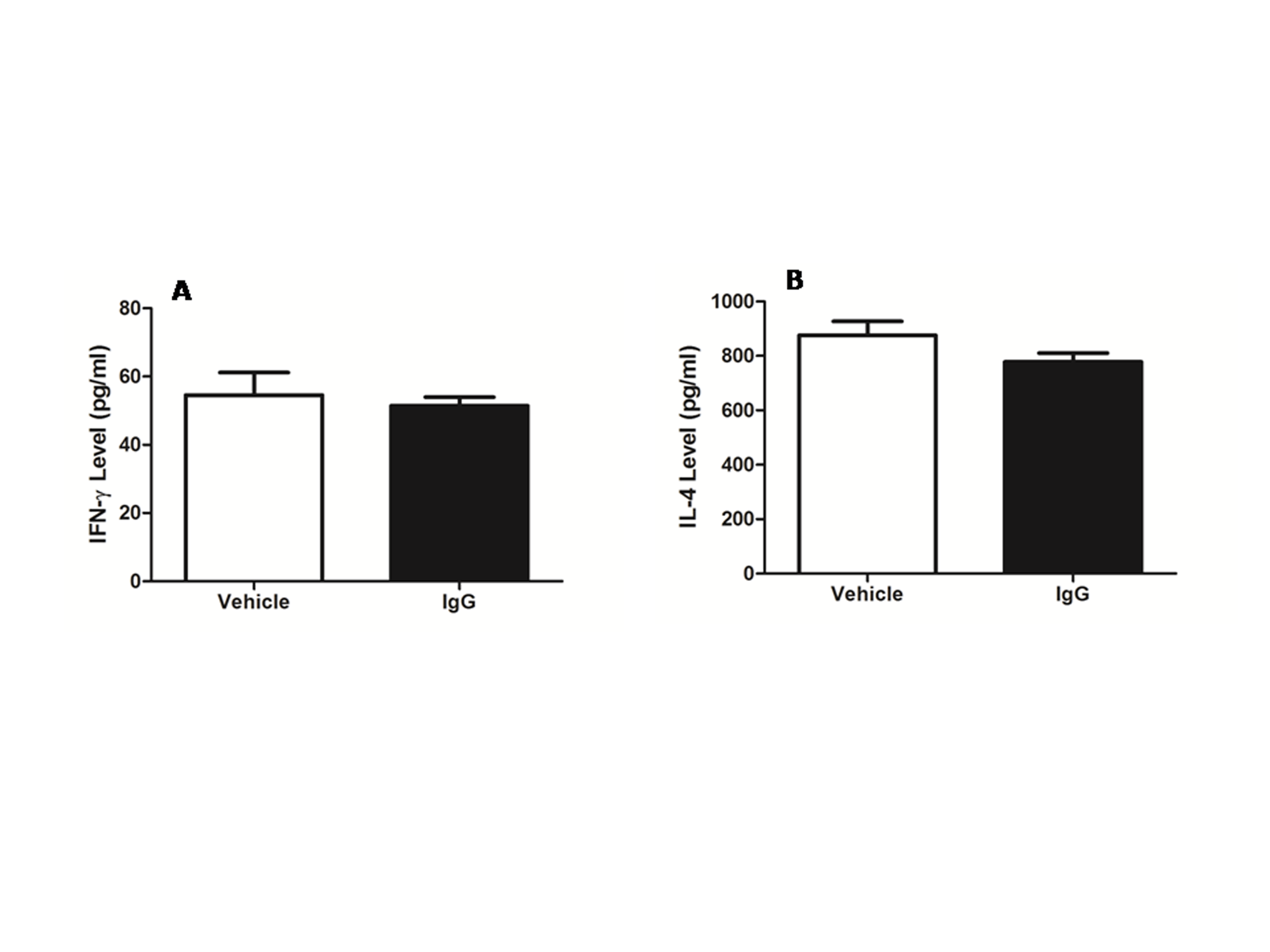

Supplement: Figure S8 — The IgGs isolated from healthy subjects revealed no effect on the levels of IFN-γ and IL-4 in CD3+T cells. n = 6 per group. Data are presented as means ± SD of 3 independent experiments. (TIF) [file pone.0052911.s008.tif]

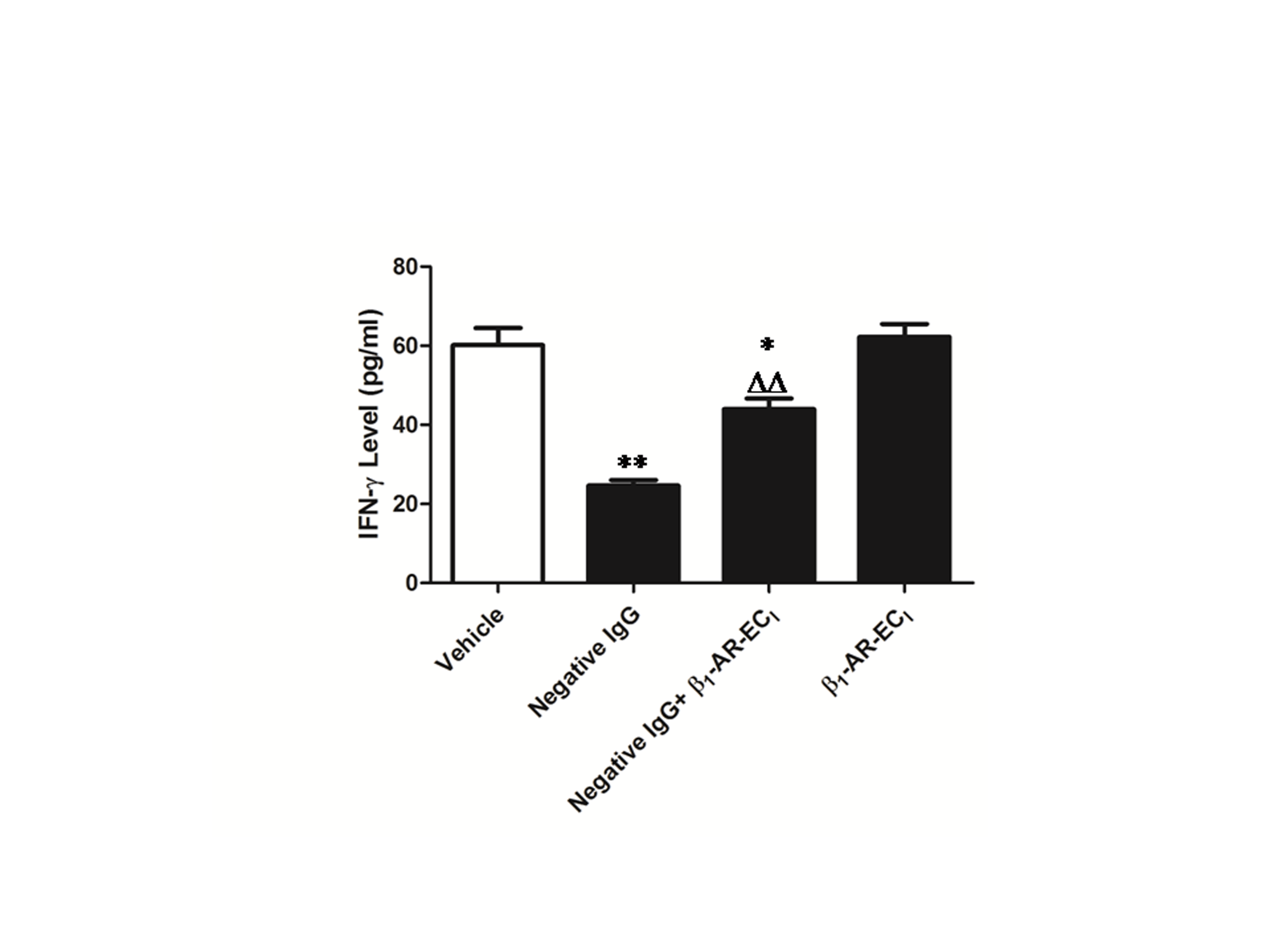

Supplement: Figure S9 — The reduce of IFN-γ induced by β1-AA-negative IgG was partially blocked by β1-AR-ECI. β1-AA-negative IgG (25 µg/ml) and the peptide corresponding to the sequence of the first extracellular loop of the human β1-AR (β1-AR-ECI, 1 µmol/l) co-incubated for 1 h at 37°C, supernatants were then collected to treat CD3+T lymphocytes, and finally IFN-γ level was analyzed by ELISA. **p < 0.01, *p < 0.05 versus. vehicle group; ΔΔ p<0.01 versus negative IgG group. n = 6 per group. Data are presented as means ± SD of 3 independent experiments. (TIF) [file pone.0052911.s009.tif]
